# Full-Term and Preterm Newborns Differ More Significantly in Photoplethysmographic Waveform Variability than Heart Rate Variability

**DOI:** 10.3390/life14060675

**Published:** 2024-05-24

**Authors:** Anton R. Kiselev, Elena N. Mureeva, Viktoria V. Skazkina, Olga S. Panina, Anatoly S. Karavaev, Yuri V. Chernenkov

**Affiliations:** 1 Coordinating Center for Fundamental Research, National Medical Research Center for Therapy and Preventive Medicine, 101990 Moscow, Russia; 2 Department of Pediatrics and Neonatology, Saratov State Medical University, 410012 Saratov, Russia; 3 Department of Dynamic Modeling and Biomedical Engineering, Saratov State University, 410012 Saratov, Russia

**Keywords:** newborns, cardiovascular autonomic regulation, photoplethysmographic waveform variability, heart rate variability

## Abstract

Background: Features of cardiovascular autonomic regulation in infants are poorly studied compared with adults. However, the clinical significance of autonomic dysfunction in infants is very high. The goal of our research was to study the temporal and frequency-dependent features, as well as low-frequency synchronization in cardiovascular autonomic regulation in full-term vs. preterm newborns, based on the analysis of their heart rate variability (HRV) and photoplethysmographic waveform variability (PPGV). Methods: The study included three groups of newborns: 64 full-term newborns (with a gestational age at birth of 37–40 weeks) with a physiological course of the neonatal adaptation; 23 full-term newborns (with a gestational age at birth of 37–40 weeks) with a pathological course of the neonatal adaptation; and 17 preterm newborns (with a postconceptional age of 34 weeks or more). We conducted spectral analysis of HRV and PPGV, along with an assessment of the synchronization strength between low-frequency oscillations in HRV and in PPGV (synchronization index). We employed several options for the boundaries of the high-frequency (HF) band: 0.15–0.40 Hz, 0.2–2 Hz, 0.15–0.8 Hz, and 0.24–1.04 Hz. Results: Preterm newborns had higher heart rate, RMSSD, and PNN50 values relative to both groups of full-term newborns. Values of SDNN index and synchronization index (S index) were similar in all groups of newborns. Differences in frequency domain indices of HRV between groups of newborns depended on the considered options of HF band boundaries. Values of frequency domain indices of PPGV demonstrated similar differences between groups, regardless of the boundaries of considered options of HF bands and the location of PPG signal recording (forehead or leg). An increase in sympathetic influences on peripheral blood flow and a decrease in respiratory influences were observed along the following gradient: healthy full-term newborns → preterm newborns → full-term newborns with pathology. Conclusions: Differences in frequency domain indices of autonomic regulation between the studied groups of newborns depended on the boundaries of the considered options of the HF band. Frequency domain indices of PPGV revealed significantly more pronounced differences between groups of newborns than analogous HRV indicators. An increase in sympathetic influences on peripheral blood flow and a decrease in respiratory influences were observed along the following gradient: healthy full-term newborns → preterm newborns → full-term newborns with pathology.

## 1. Background

The usefulness and accessibility of the assessment of heart rate or photoplethysmogram (PPG) in neonatology are based on the ease of recording the necessary biological signals using various devices or systems. Indeed, chronotropic regulation of the heart, conventionally assessed by analyzing heart rate variability (HRV) from an electrocardiogram (ECG), is critical for the newborn [1].

Analysis of HRV is a generally accepted method for studying the autonomic regulation of the cardiovascular system. In 1996, the European Society of Cardiology and the North American Society of Pacing and Electrophysiology developed recommendations for the physiological interpretation and clinical use of basic HRV indicators [2]. Various time and frequency domain indices are used to analyze the HRV. An overview of HRV metrics is detailed in a number of reviews [3,4,5,6,7]. It uses time domain indices such as the standard deviation of normal-to-normal (NN) intervals (the time elapsing between two consecutive R waves in the ECG with normal sinus rhythm) (abbreviated as SDNN), the percentage of consecutive NN intervals differing by more than 50 ms (PNN50), the square root of the mean sum of squared differences between adjacent NN intervals (RMSSD), etc. There is an opinion that the global behavior of the HRV can be assessed from the value of the SDNN parameter, and the parasympathetic activity can be assessed according to PNN50 and RMSSD [2,4,8]. For frequency-domain HRV indices, such as the power spectral density of low-frequency (LF) and high-frequency (HF) bands of the HRV spectrum, absolute power components (in ms^2^) and relative power components (in percent) are used to measure. According to the classical physiological interpretation, the HF band of HRV spectrum reflects primarily the respiratory and parasympathetic influences on the heart rhythm; the LF band reflects mainly baroreflex and sympathetic influences, but parasympathetic tone also affects its formation. Sometimes the ratio of low frequencies to high frequencies (LF/HF) that reflects the level of vagosympathetic balance is also being calculated [2,4,8].

The PPG signal can also be used to assess cardiac chronotropic regulation through pulse rate variability (PRV) determined by the PPG [9]. Furthermore, in addition to PRV, information about other physiological processes (blood pressure [10], systemic vascular resistance [11], arterial tone and stiffness [12,13], and vasomotor response [14]) can be obtained from the PPG signal by analyzing photoplethysmographic waveform variability (PPGV).

In adults, the basic principles of the functional organization of cardiovascular autonomic regulation are known, such as cardiorespiratory interaction [15,16,17], strengthening of the baroreflex [18,19,20], phase synchronization or coherence in the cardiovascular system (CVS) [17,20,21], and central neural influence [22,23]. Analysis of spontaneous oscillations in heart rate, blood pressure, and peripheral blood flow is a popular approach to studying the frequency-dependent features of autonomic regulation of the cardiovascular system. Many studies have also focused on blood pressure variability (BPV) [24] and PPGV [25] to assess autonomic regulation. Moreover, Kamshilin et al. reason about the close relationship between reflection-mode PPG and blood pressure signals based on their own physiologic model [26]. Previously, while studying nonlinear interactions in the autonomic regulation of the cardiovascular system, we identified the synchronization of low-frequency (LF) oscillations in HRV and PPGV [21], proposed a method for its quantitative assessment [27], and demonstrated the prospect of clinical application [27,28]. The accumulated scientific knowledge provided the basis for mathematical modeling of the autonomic regulation of the cardiovascular system [29,30,31,32,33,34].

Frequency-dependent features of the autonomic regulation of the cardiovascular system in newborns were poorly studied compared with adults. At the same time, the clinical significance of autonomic dysfunction in infants is very high. There is a known association between changes in cardiorespiratory coupling and sudden infant death syndrome [35,36]. Dimitrijević et al. reported that HRV indicators with abnormal general movements can be used to predict neurodevelopmental outcomes in preterm infants [37]. Data on infant HRV, BPV, and PPGV are limited and have significant inconsistencies in the methods of analysis used [38,39,40,41]. Previously, we attempted to investigate the nonlinear interactions between low-frequency oscillations in HRV and PPGV based on analyses of synchronization, coherence, and cross-recurrence [42,43].

The goal of our research was to study the temporal and frequency-dependent features as well as low-frequency synchronization in cardiovascular autonomic regulation in full-term vs. preterm newborns based on the analysis of their HRV and PPGV.

## 2. Materials and Methods

### 2.1. Study Subjects

The study was carried out at the Department of Pediatrics and Neonatology, Saratov State Medical University (Saratov, Russia).

The study included three groups of newborns:

(i). Group 1 comprises 64 full-term newborns (gestational age at birth of 37–40 weeks) with a physiological course of their neonatal adaptation, chosen on the basis of the healthy anamneses of their mothers and a normal birth. The identification of illnesses of any kind constituted a motive for disqualification.
(ii) Group 2, consisting of 23 full-term newborns (gestational age at birth of 37–40 weeks), with a pathological course of their neonatal adaptation. These children were placed in the department of pathology for preterm newborns and neonates with various diseases of the perinatal period, viz., respiratory disorders (respiratory distress syndrome, birth asphyxia, congenital pneumonia, aspiration syndrome), intrauterine growth restriction, pathology of the central nervous system, neonatal jaundice, and hemolytic disease of the newborn. Synchronized recording of ECG, PPG, and respiration in this group of newborns was carried out after stabilization of their condition.
(iii) Group 3, encompassing 17 preterm infants (postconceptional age of 34 weeks or more).

### 2.2. Recordings of Biological Signals

Synchronized recordings of ECG, PPGs, and respiration were performed for 15 min in the afternoon (3:00–4:00 p.m.) in all newborns on the third day after birth. Infrared PPG sensors for reflected light were applied to the heel and forehead of the newborn. The respiratory recursion sensor made it possible to register thoracic and abdominal respiratory effort. Signals were recorded during feeding, which allowed obtaining data in a state of wakefulness.

The above recordings were performed using a multichannel biological signal recorder, Reactor-T (Medicom-MTD, Taganrog, Russia). The cutoff frequency for a high-pass filter when recording all signals was 0.016 samples per second (sps). The signals were recorded in a quiet room with a controlled temperature. All signals were sampled at 250 sps and digitized at 14 bits.

**Figure 1:**
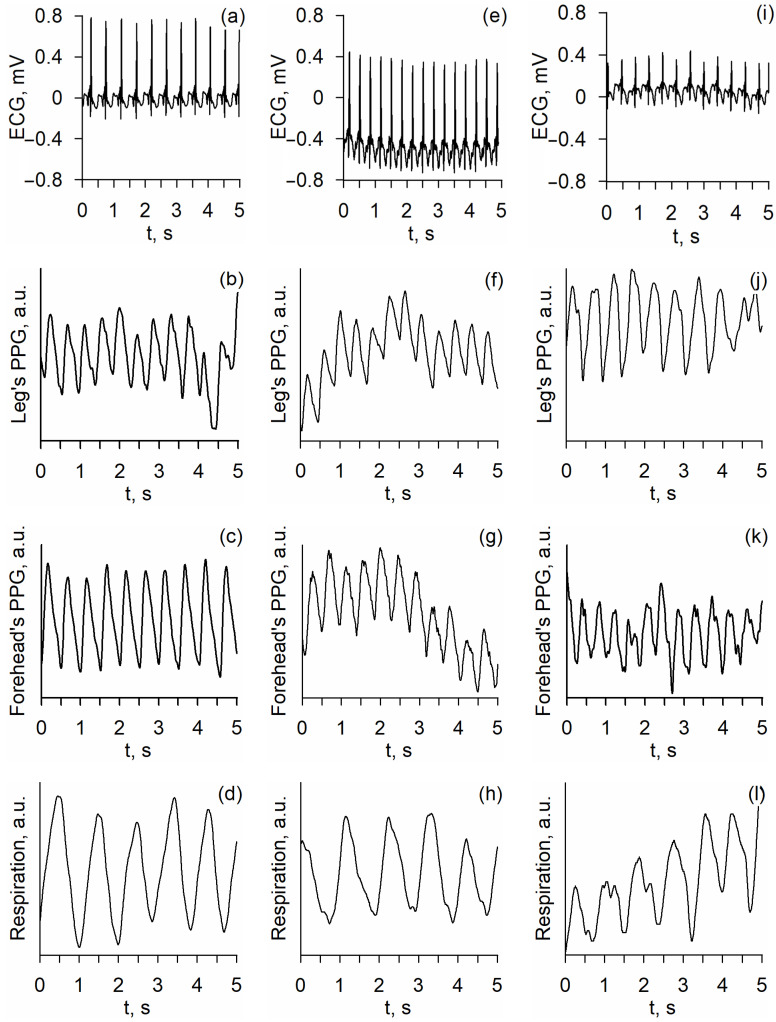
Examples of ECG (**a**,**e**,**i**), forehead PPG (**c**,**g**,**k**), leg PPG (**b**,**f**,**j**), and respiratory signals (**d**,**h**,**l**) in healthy full-term newborns (Group 1; (**a**–**d**)), full-term newborns with pathology (Group 2; (**e**–**h**)), and preterm newborns (Group 3; (**i**–**l**)). Note: ECG—electrocardiogram; PPG—photoplethysmogram.

Examples of biological signals in groups under research (healthy full-term newborns, full-term newborns with pathology, and preterm newborns) are shown in Figure 1.

Only records without artifacts and considerable trends were used for the final analysis. For ECG, records with normal sinus rhythm were used for further preprocessing.

### 2.3. Data Preprocessing

**Figure 2:**
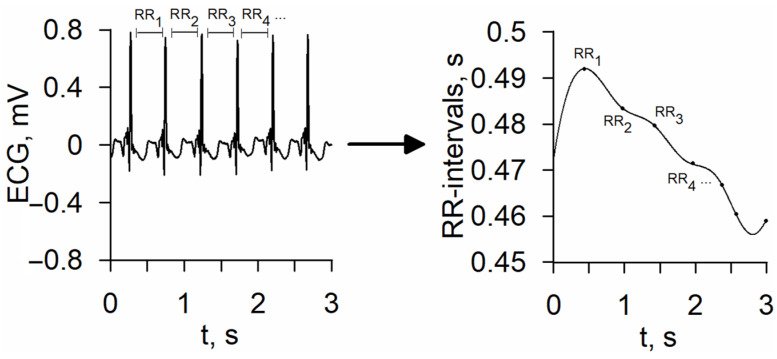
Extraction of RR-interval sequence from ECG. Note: ECG—electrocardiogram.

The NN interval sequence (the time elapsing between two consecutive R waves in the ECG with normal sinus rhythm) was extracted from the ECG in order to analyze HRV (Figure 2). Due to heart rate variations, the NN interval measurements are non-equidistant in time. To transform a non-equidistant signal to a equidistant one, we approximated it by cubic splines, low-pass filtering (cutoff 0.5 Hz), and then resampling at regular intervals with a 4 Hz sampling rate (in accordance with R.M. Baevsky et al. [8]). The resulting equidistant sequence of NN intervals was used for further processing.

PPG and respiratory signals were not preprocessed.

### 2.4. Analysis of Heart Rate Variability

HRV was analyzed in the time and frequency domains.

Spectral analysis of HRV was carried out according to the methodological guidelines adopted in Russia [8,44,45]. Data segments of 300 s were used for spectral analysis. Linear trends were removed, and power spectral density was estimated with the fast Fourier transformation-based Welch algorithm using segments of 256 data points with 50% overlapping and a Hanning window.

The following boundaries are conventionally used in the spectral analysis of HRV in adults: low-frequency (LF) band, 0.04–0.15 Hz, often considered to represent sympathetic activity and baroreflex; and high-frequency (HF) band, 0.15–0.40 Hz, considered to represent parasympathetic activity and the effect of respiration [2]. However, the boundaries of the HF band are actively debated given the extensive biological variability of respiratory rates; in children and in some conditions, respiratory rates may exceed the specified boundaries [46]. To resolve this issue, we employed several options for HF band boundaries:

(i) HF1 band: 0.15–0.40 Hz, conventionally used for adults [2,8];
(ii) HF2 band: 0.2–2 Hz, frequently used when studying children [41];
(iii) HF3 band: 0.15–0.8 Hz, proposed for children by Alkon et al. [47,48];
(iv) HF4 band: 0.24–1.04 Hz, recommended for small children by Lackner et al. [49].

We calculated the following indices of HRV:

1. mean heart rate in beats per minute (named as HR, bpm);
2. standard deviations of normal-to-normal (NN) intervals (the time elapsing between two consecutive R waves in the ECG with normal sinus rhythm) (named as SDNN, ms);
3. square root of the mean squared differences of successive normal intervals (root mean square successive difference abbreviated as RMSSD, ms);
4. percentage of adjacent NN intervals differing from each other by more than 50 ms (named as PNN50, %);
5. total power spectral density (named as TP) of HRV spectrum measured in ms^2^ and integrated in 0–0.4 Hz band (hereinafter named as TP1, ms^2^), 0–0.15 + 0.2–2 Hz band (named as TP2, ms^2^), 0–0.8 Hz band (named as TP3, ms^2^), and 0–0.15 + 0.24–1.04 Hz band (named as TP4, ms^2^);
6. power spectral density of LF band (0.04–0.15 Hz) in HRV spectrum measured in ms^2^ (named as LF, ms^2^);
7. power spectral density of HF band in HRV spectrum measured in ms^2^ and integrated our accepted boundary options: 0.15–0.40 Hz band (named as HF1, ms^2^), 0.2–2 Hz band (named as HF2, ms^2^), 0.15–0.8 Hz band (named as HF3, ms^2^), and 0.24–1.04 Hz band (named as HF4, ms^2^);
8. the ratio of LF to TP as a percentage (for TP1, named as LF1%; for TP2, named as LF2%; for TP3, named as LF3%; and for TP4, named as LF4%);
9. the ratio of HF to TP as a percentage (for HF1 and TP1 named as HF1%; for HF2 and TP2 named as HF2%; for HF3 and TP3 named as HF3%; for HF4 and TP4 named as HF4%);
10. the ratio of LF to HF (for HF1 named as LF/HF1; for HF2 named as LF/HF2; for HF3 named as LF/HF3; and for HF4 named as LF/HF4).

The VLF range was not included in our analysis to avoid questionable results because we registered short-time ECG records [2].

### 2.5. Spectral Analysis of Photoplethysmographic Waveform Variability

PPGV power spectra were calculated directly from the PPG signal. The power spectrum was estimated using Welch’s method [50] in two-minute windows with a one-minute shift. A critical power value was calculated, above which the spectral components were considered statistically significant (*p* = 0.05). To do so, using surrogate data, we tested the statistical hypothesis of normal band-limited noise (0.04–2 Hz). Before the study, we compared power spectral density estimates at different window lengths. As a result, we have chosen the above parameters because they (a) correspond to the official recommendations adopted in Russia for the spectral assessment of HRV signals [8] and (b) yield satisfactory results in estimating the power in the frequency band when comparing groups by PPG signals, although promising methods of data analysis based on parametric spectral estimation or nonlinear filtering (e.g., via empirical mode decomposition) are known [51].

Then, using an approach similar to that used for the analysis of HRV [2,8] and the options we used for the HRV boundaries of the HF band, we calculated the following spectral PPGV indices for these spectra:

(i) the ratio (as a percentage) of the power density of the LF band (LF: 0.04–0.15 Hz) to the total power density (TP) of the PPG spectrum integrated in the 0–0.4 Hz band (named as LF1%), 0–0.15 + 0.2–2 Hz band (named as LF2%), 0–0.8 Hz band (named as LF3%), and 0–0.15 + 0.24–1.04 Hz band (named as LF4%);
(ii) the ratio (as a percentage) of the power density of HF band (HF1: 0.15–0.40 Hz, HF2: 0.2–2 Hz, HF3: 0.15–0.8 Hz, HF4: 0.24–1.04 Hz) to TP (TP1: 0–0.4 Hz, TP2: 0–0.15 + 0.2–2 Hz, TP3: 0–0.8 Hz, TP4: 0–0.15 + 0.24–1.04 Hz) of the PPG spectrum (named as LF1%, LF2%, LF3%, and LF4%, respectively);
(iii) the ratio of LF to HF (for HF1 named as LF/HF1, for HF2 named as LF/HF2, for HF3 named as LF/HF3, and for HF4 named as LF/HF4).

One of the problems we have encountered with PPG is the difficulty in interpreting the absolute values of PPG signals. The output signal of a PPG sensor is proportional to an unknown coefficient that depends on a number of factors, such as the optical characteristics of the subject’s skin, blood pressure values, sensor placement, electrical and optical characteristics of the sensor, and room illumination and temperature. The absolute values of the PPG waveform were measured in arbitrary units (a.u.), which were defined as the proportion of a discrete sample from the PPG waveform of the optical sensor output signal. Since the proportionality coefficient between volumetric blood flow and a.u. is unknown, the interpretation of the absolute values of low-frequency, high-frequency, and total powers of the PPG spectrum is rather difficult. We do not use these spectral indices in this paper. However, the dimensionless measurements (LF/HF, LF%, and HF%) are applicable. We have previously successfully used the described approach to PPGV analysis in studies involving adults [52].

### 2.6. Assessment of Synchronization between the Low-Frequency Oscillations in Heart Rate Variability and Photoplethysmographic Waveform Variability

To measure the strength of synchronization between low-frequency oscillations in HRV and PPGV, the overall percentage of phase synchronization (named the S index) was introduced [27]. This index is calculated for a pair of signals, PPG and NN intervals, extracted from the ECG. The calculation of the S index consists of several stages: the PPG and NN interval signals are filtered with a 0.05–0.15 Hz bandpass filter; oscillation phases are calculated from filtered signals; the difference between the resulting phases is calculated; an automated algorithm for detecting horizontal sections of signal phase differences was applied; horizontal sections correspond to frequency synchronization intervals between signals; and the total duration of the horizontal sections is calculated and divided by the total duration of the signals to estimate the S index. A detailed description of the S index calculation method is presented in our previous article [27].

### 2.7. Statistical Data Processing

Statistical analysis of the results included checking the compliance of numerical data with the normal distribution law using the Shapiro–Wilk test. Since most variables were not normally distributed, further analysis was performed using nonparametric statistical methods. Data are presented as a median with lower and upper quartiles, Me (LQ, UQ). We used Kruskal–Wallis ANOVA with post hoc comparisons of mean ranks for multiple comparisons of continuous variables between the studied groups. The results were considered statistically significant at *p* < 0.05.

## 3. Results

**Figure 3:**
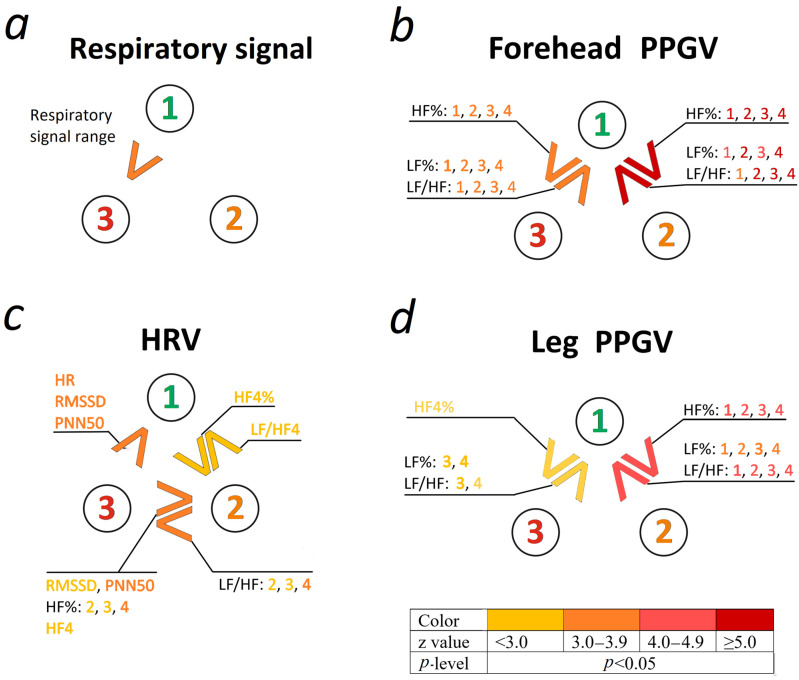
Comparison of the studied groups of newborns based on HRV (**c**), PPGV (assessed on the forehead and leg) (**b**,**d**), and respiratory signal (**a**). Note: 1, Group 1 (healthy full-term newborns); 2, Group 2 (full-term newborns with pathology); 3, Group 3 (preterm newborns); “greater than” (>) and “less than” (<) signs are shown in different colors based on the strength of differences calculated using the z score and *p*-levels (post hoc analysis). See the color gradation chart in the figure; for each sign “more than” (>) and “less than” (<), there is a list of indicators that exhibited statistically significant differences in the corresponding direction; indicators with similar magnitudes in the groups are not shown in the figure; their values can be found in Table 1. PPGV—photoplethysmographic waveform variability; HRV—heart rate variability. Time domain indices of HRV: HR—mean heart rate; SDNN—standard deviation of the NN interval (the time elapsing between two consecutive R waves in the electrocardiogram with normal sinus rhythm); RMSSD—square root of the mean squared differences of successive NN intervals; PNN50—proportion derived by dividing NN50, the number of interval differences of successive NN intervals greater than 50 ms, by the total number of NN intervals. Frequency domain indices: LF—power spectral density of low-frequency band in spectrum measured in ms^2^; TP1…4—total power spectral density of spectrum measured in ms^2^ and integrated in 0–0.4 Hz band (named as TP1), 0–0.15 + 0.2–2 Hz band (named as TP2), 0–0.8 Hz band (named as TP3), and 0–0.15 + 0.24–1.04 Hz band (named as TP4); HF1…4—power spectral density of high-frequency band in spectrum measured in ms^2^ and integrated our accepted boundary options: 0.15–0.40 Hz band (named as HF1), 0.2–2 Hz band (named as HF2), 0.15–0.8 Hz band (named as HF3), and 0.24–1.04 Hz band (named as HF4); LF1…4%—low-frequency band in percentage of total spectral power (TP1…4, consequently); HF1…4%—high-frequency band (HF1…4, consequently) in percentage of total spectral power (TP1…4, consequently); LF/HF1...4—the ratio of low-frequency band to high-frequency band (HF1…4, consequently).

Results of comparative analysis of HRV, PPGV (evaluated on the forehead and leg), respiratory signal and S index in groups of newborns are presented in Figure 3 and Table 1 (descriptive statistics, results of the Kruskal–Wallis test with post hoc comparisons), and Supplementary Materials (a color gradation chart based on strength differences calculated from the z score and *p*-level in post hoc analyses).

Preterm newborns (Group 3) had statistically significantly higher values of heart rate and two temporal domain indices of HRV (RMSSD and PNN50) relative to both groups of full-term newborns. SDNN and the S index had similar values in all three groups of newborns.

Differences in frequency domain indices of HRV between the studied groups of newborns depended on the considered boundaries of the HF band. The groups did not differ in any HF1-related parameters. When using the HF2 and HF4 bands, preterm newborns (Group 3) were characterized by higher values of HF (in ms^2^) and HF% and lower values of LF/HF (vs. healthy full-term newborns or full-term infants with pathology). When the HF3 band was considered, preterm infants (Group 3) differed only from full-term newborns with pathology (Group 2) in HF% and LF/HF. LF (in ms^2^) was similar in all three groups of newborns.

Frequency domain indices of PPGV showed the same type of differences between groups, regardless of the considered options of HF band boundaries and the location of PPG signal recording (forehead PPG or leg PPG). Preterm newborns (Group 3) and full-term newborns with pathology (Group 2) were characterized by unidirectional differences from healthy full-term newborns (Group 1). Specifically, LF% and LF/HF exhibited higher values, while HF% demonstrated lower values. The direction of change (increase or decrease) in the spectral parameters of PPGV between groups was observed along the following gradient: healthy full-term newborns (Group 1) → preterm newborns (Group 3) → full-term newborns with pathology (Group 2).

## 4. Discussion

According to the results of our study, temporal and frequency domain indices of HRV, frequency domain indices of PPGV, and S index demonstrated differences between the studied groups of newborns. This may characterize differences in changes in autonomic regulation of heart rate and peripheral blood flow (assessed by the PPG signal) depending on the gestational age at birth and the presence of neonatal pathology.

The methodology for studying frequency-dependent features of cardiovascular autonomic regulation in newborns is a crucial problem of physiology, considering the diversity of opinions and positions of researchers regarding the names of indicators and the boundaries of frequency bands [41]. However, more than a quarter of researchers use the following boundaries of the LF range of HRV: 0.04–0.15 Hz [41], which complies with the recommendations for adults [2,8]. For the HF range, the most common boundaries are 0.2–2 Hz [41], which differs from the recommended boundaries for adults of 0.15–0.4 Hz [2,8]. Only approximately 8% of studies use the boundaries for both the LF and HF bands of the HRV spectrum in newborns, fully complying with recommendations for adults [41]. The rationale for using non-standard boundaries for LF and HF bands is the differences in respiratory and heart rates between newborns and adults [53]. The lack of a unified position on the methodology of frequency domain analysis of HRV makes it difficult to compare the results of different authors and their physiological interpretation.

Overall, our study obtained similar results for comparing frequency domain indices of HRV and PPGV between groups of newborns for all considered options of HF band boundaries. However, the HRV and PPGV indicators associated with the HF1 band and HF3 band demonstrated less significant differences (up to their absence) vs. similar groups of indicators associated with HF2 and HF4.

It is important to emphasize that the studied groups of newborns differed significantly from each other in all PPGV indices, regardless of the location of PPG recording (leg or forehead), while differences in HRV characteristics were noted only for preterm newborns (vs. healthy full-term newborns or full-term newborns with pathology) for some indicators (HR, RMSSD, PNN50, HF2, HF2%, LF/HF2, HF3%, LF/HF3, HF4, HF4%, and LF/HF4). Note that RMSSD is most appropriate and least problematic unless the focus is on highly accurate heart rate variability analysis [49]. The strength of synchronization between the LF oscillations in HRV and PPGV was similar in all groups of newborns.

According to our data, only preterm newborns exhibited any significant changes in autonomic influences on heart functioning (based on analysis of HRV indicators). However, the physiological interpretation of the results is equivocal. On the one hand, higher heart rate values implied increased sympathicotonia in preterm newborns. On the other hand, there were signs of increased respiratory and parasympathetic influences in the form of higher values of indicators characterizing the HF band of the HRV spectrum [2], PNN50 [3,54], and RMSSD [3,55].

Frequency domain indices of PPGV revealed much more pronounced differences between groups of newborns than equivalent HRV indicators. Usually, the activity in the HF band of the PPG power spectrum is related to the mechanical consequence of respiration [56,57,58], whereas the activity in the LF band is associated with the regulation of peripheral vascular tone [56,59]. It is noteworthy that in our study, an increase in sympathetic influences on peripheral blood flow (based on indicators characterizing the LF band of the PPG spectrum [56,59]) and a decrease in respiratory effects (based on indicators characterizing the HF band of the PPG spectrum [56,59]) were observed along the following sequence: healthy full-term newborns → preterm newborns → full-term newborns with pathology. These changes accordingly affect the LF/HF ratio.

The most common opinion regarding the nature of the LF/HF ratio is its association with the ratio of sympathetic to parasympathetic activity [60]. However, such reasoning has serious limitations. In particular, a number of authors doubted the point of view that the LF/HF ratio measures cardiac sympathovagal balance [61]. First, LF power is not just an index of sympathetic activity. Half of the variability in this frequency band is due to parasympathetic activity, and a smaller proportion is caused by unspecified factors. Second, sympathetic-parasympathetic interactions are complex, nonlinear, and frequently nonreciprocal. Third, confounding caused by respiratory mechanics and resting heart rate creates uncertainty in terms of parasympathetic and sympathetic contributions to the LF/HF ratio during the measurement period.

It is acknowledged that the LF/HF ratio of HRV in human adults is close to 1 during the resting supine position [2]. In healthy full-term newborns in our study, this indicator was on average 1.5–3 times greater depending on the HF band boundaries used in the calculation, which indicates the predominance of sympathetic and baroreflex impacts on cardiac function in newborns vs. adults (according to one of our previous studies, the LF/HF ratio of HRV in human adults aged 27 ± 3 years was 0.8 (0.5, 1.3) [25]). With regard to the regulation of peripheral blood flow assessed by the PPG signal in newborns, there is normally a significant predominance of respiratory influences over sympathetic effects, relative to adults, according to our previous data [25,52]. However, in previous studies, the values of this indicator in adults differed substantially in different age groups and locations of the PPG signal recording. E.g., for finger PPG in healthy men aged 48.8 ± 4.5 years, the LF/HF ratio of PPGV was 0.60 (0.23, 4.79) [52], whereas in men aged 27 ± 3 years it was 8.2 (5.5, 11.2) [25]. In the latter group, the LF/HF ratio was also assessed when recording PPG from the earlobe as 13.5 (5.2, 21.5) [25].

It is also well known that the LF/HF ratio of the HRV spectrum progressively decreases with postnatal age, implying an increase in the parasympathetic effect on cardiac chronotropic regulation [62,63,64]. In our study, we observed significantly lower values of this indicator for the PPG signal on the third day after birth in healthy full-term newborns vs. full-term infants with pathology and vs. preterm newborns. HRV analysis did not reveal similar differences.

A comparative analysis of the absolute powers of the HRV spectral ranges in newborns vs. adults is difficult, given the lack of a unified position on the choice of the boundaries of the HF band and TP.

The S index, previously proposed by us, was 1.5–2 times lower in newborns of all studied groups than in healthy adult men, according to our previous studies: 38.1% (30.3, 44.2) [25]. Perhaps this may characterize the functional immaturity of the systemic organization in the autonomic regulation of blood circulation.

## 5. Conclusions

Differences in frequency domain indices of autonomic regulation between the studied groups of newborns depended on the boundaries of the considered options of the HF band. Frequency domain indices of PPGV revealed significantly more pronounced differences between groups of newborns than analogous HRV indicators.

An increase in sympathetic influences on peripheral blood flow and a decrease in respiratory influences were observed along the following gradient: healthy full-term newborns → preterm newborns → full-term newborns with pathology.

## 6. Limitations

It should be noted that in this work we have analyzed data from a relatively small number of newborns, and therefore it is necessary to perform a larger study. At this stage of our work, the mechanistic aspects of the identified associations were not investigated. Among these, for example, are sex, birth weight, Apgar score, clinical interventions, and other indicators that can affect autonomic control of vascular tone and heart rate.

Also, the results obtained in the course of the work do not answer the question of whether the observed differences in frequency domain indices of cardiovascular autonomic regulation have an effect on the prognosis of newborns.

Signals were recorded during feeding that were necessary to exclude the known effects of falling asleep and sleeping on cardiovascular autonomic regulation [65,66,67]. We set out to study autonomic regulation in wakefulness. Careful screening of recordings for movement artifacts also contributed to the difficulty in recruiting large groups of subjects.

## Figures and Tables

**Table 1:** Assessment of HRV, PPGV, respiration and S index in the studied groups of newborns.

Parameters	Healthy Full-Term Newborns (Group 1) (n = 64)	Full-Term Newborns with Pathology (Group 2) (n = 23)	Preterm Newborns (Group 3) (n = 17)	Kruskal-Wallis Test
HR, bpm	131.1 (122.0, 139.7)	137.6 (126.1, 148.1) z_1_ = 1.4, *p* = 0.469	151.8 (141.6, 160.1) z_1_ = 3.9, *p* < 0.001 z_2_ = 2.4, *p* = 0.056	H = 15.8, *p* = 0.004
Respiratory frequency, Hz	1.03 (0.84, 1.22)	0.99 (0.83, 1.19)	1.12 (0.87, 1.31)	H = 0.8, *p* = 0.683
Respiratory signal range	1.9 (1.3, 2.5)	1.6 (1.2, 2.1) z_1_ = 1.4, *p* = 0.491	1.1 (0.9, 1.6) z_1_ = 3.2, *p* = 0.005 z_1_ = 1.7, *p* = 0.283	H = 10.4, *p* = 0.006
S index (HRV-PPGV forehead), %	15.9 (11.9, 20.3)	17.2 (15.4, 20.3)	17.5 (15.6, 21.6)	H = 1.86, *p* = 0.394
S index (HRV-PPGV leg), %	15.5 (11.1, 20.1)	18.5 (14.1, 21.8)	14.4 (12.7, 16.2)	H = 3.47, *p* = 0.177
HRV				
SDNN, ms	31.8 (21.1, 45.6)	27.7 (16.9, 35.9)	33.9 (31.9, 38.2)	H = 2.9, *p* = 0.237
RMSSD	15.2 (10.3, 36.3)	13.3 (9.2, 21.1) z_1_ = 1.3, *p* = 0.545	26.6 (22.5, 48.3) z_1_ = 3.9, *p* = 0.004 z_2_ = 2.4, *p* = 0.029	H = 10.7, *p* = 0.005
PNN50	0.99 (0.01, 4.65)	0.53 (0.01, 2.97) z_1_ = 0.4, *p* = 0.975	7.65 (3.26, 16.43) z_1_ = 3.2, *p* = 0.004 z_2_ = 3.1, *p* = 0.006	H = 12.3, *p* = 0.002
LF, ms^2^	123.8 (69.1, 289.7)	123.2 (88.7, 220.7)	137.0 (96.8, 184.7)	H = 0.01, *p* = 0.997
TP1, ms^2^	446.6 (256.6, 876.3)	488.3 (383.5, 700.1)	392.5 (353.2, 499.6)	H = 0.4, *p* = 0.813
HF1, ms^2^	49.9 (19.3, 102.3)	56.5 (26.7, 86.0)	45.1 (40.1, 106.8)	H = 0.5, *p* = 0.786
LF1%	28.5 (23.8, 35.3)	27.0 (22.2, 39.9)	34.0 (28.6, 35.4)	H = 2.2, *p* = 0.336
HF1%	11.1 (6.8, 22.2)	10.5 (5.9, 15.5)	15.4 (10.2, 25.5)	H = 3.2, *p* = 0.199
LF/HF1	2.9 (1.6, 4.0)	3.4 (2.1, 4.1)	2.4 (1.3, 2.7)	H = 3.6, *p* = 0.166
TP2, ms^2^	568.9 (288.1, 1096.4)	595.9 (442.7, 782.7)	518.4 (438.0, 708.9)	H = 0.1, *p* = 0.941
HF2, ms^2^	116.6 (48.5, 353.0)	99.1 (51.4, 175.9)	194.2 (126.0, 336.7)	H = 4.4, *p* = 0.111
LF2%	22.8 (18.1, 31.2)	24.4 (20.2, 36.6)	22.5 (20.8, 26.7)	H = 1.0, *p* = 0.601
HF2%	21.3 (13.8, 45.3)	18.0 (11.2, 26.9) z_1_ = 1.5, *p* = 0.375	38.4 (25.3, 47.5) z_1_ = 2.1, *p* = 0.120 z_2_ = 2.9, *p* = 0.011	H = 8.4, *p* = 0.015
LF/HF2	1.2 (0.5, 2.1)	1.7 (0.8, 2.7) z_1_ = 1.6, *p* = 0.354	0,69 (0,55, 0,80) z_1_ = 2.0, *p* = 0.148 z_2_ = 2.9, *p* = 0.013	H = 8.1, *p* = 0.017
TP3, ms^2^	540.70 (280.20, 1013.2)	561.95 (430.45, 759.25)	454.50 (414.00, 644.30)	H = 0.03 *p* = 0.985
HF3, ms^2^	76.90 (31.30, 268.30)	91.85 (41.25, 138.75)	122.70 (80.60, 266.20)	H = 2.24 *p* = 0.326
LF3%	25.40 (20.10, 32.50)	26.90 (21.00, 37.95)	25.30 (23.50, 29.40)	H = 0.24 *p* = 0.886
HF3%	18.20 (10.80, 35.40)	16.30 (9.35, 20.55) z_1_ = 1.40, *p* = 0.488	28.90 (18.50, 42.20) z_1_ = 1.94, *p* = 0.159 z_2_ = 2.70, *p* = 0.021	H = 7.29 *p* = 0.026
LF/HF3	1.60 (0.80, 2.50)	2.00 (1.20, 2.95) z_1_ = 1.34, *p* = 0.540	1.00 (0.70, 1.20) z_1_ = 2.05, *p* = 0.121 z_2_ = 2.76, *p* = 0.017	H = 7.65 *p* = 0.022
TP4, ms^2^	568.42 (285.10, 1062.3)	547.35 (421.71, 722.33)	483.20 (427.56, 683.94)	H = 0.15 *p* = 0.926
HF4, ms^2^	96.73 (33.13, 302.65)	60.40 (27.96, 123.61) z_1_ = 2.09, *p* = 0.110	168.52 (102.17, 311.71) z_1_ = 1.45, *p* = 0.443 z_2_ = 2.78, *p* = 0.016	H = 8.24 *p* = 0.016
LF4%	24.58 (19.42, 31.28)	26.79 (21.61, 38.91)	23.84 (21.83, 27.94)	H = 1.55 *p* = 0.461
HF4%	20.64 (12.92, 40.63)	12.92 (6.40, 22.83) z_1_ = 2.73, *p* = 0.019	35.09 (21.99, 45.58) z_1_ = 2.05, *p* = 0.122 z_2_ = 3.77, *p* < 0.001	H = 14.92 *p* < 0.001
LF/HF4	1.50 (0.61, 2.20)	2.88 (1.10, 5.28) z_1_ = 2.66, *p* = 0.023	0.82 (0.59, 1.02) z_1_ = 1.90, *p* = 0.17 z_2_ = 3.60, *p* < 0.001	H = 13.70 *p* = 0.001
Forehead PPGV				
LF1%	30.19 (22.49, 37.49)	48.10 (44.02, 54.44) z_1_ = 4.83, *p* < 0.001	46.61 (32.00, 54.07) z_1_ = 3.42, *p* = 0.002 z_2_ = 0.83, *p* = 1.000	H = 27.488 *p* < 0.001
HF1%	65.48 (53.49, 73.22)	26.60 (20.87, 35.36) z_1_ = 5.16, *p* < 0.003	30.75 (23.08, 62.97) z_1_ = 3.31, *p* = 0.003 z_2_ = 1.19, *p* = 0.700	H = 29.983 *p* < 0.001
LF/HF1	0.46 (0.31, 0.69)	2.06 (1.42, 2.52) z_1_ = 3.47, *p* < 0.001	1.61 (0.50, 2.58) z_1_ = 3.47, *p* = 0.002 z_2_ = 1.03, *p* = 0.911	H = 30.378 *p* < 0.001
LF2%	14.84 (10.82, 22.76)	43.82 (37.01, 48.71) z_1_ = 5.26, *p* < 0.001	39.81 (19.07, 50.10) z_1_ = 3.47, *p* = 0.002 z_2_ = 1.03, *p* = 0.895	H = 31.66 *p* < 0.001
HF2%	82.72 (71.61, 87.07)	32.24 (25.96, 46.39) z_1_ = 5.45, *p* < 0.001	40.48 (26.38, 78.51) z_1_ = 3.44, *p* = 0.002 z_2_ = 1.22, *p* = 0.670	H = 33.36 *p* < 0.001
LF/HF2	0.20 (0.10, 0.30)	1.40 (0.90, 1.80) z_1_ = 5.30, *p* < 0.001	1.00 (0.25, 1.95) z_1_ = 3.33, *p* = 0.003 z_2_ = 1.19, *p* = 0.697	H = 31.89 *p* < 0.001
LF3%	20.10 (15.10, 27.30)	45.90 (38.80, 50.60) z_1_ = 4.96, *p* < 0.001	43.70 (22.50, 52.80) z_1_ = 3.47, *p* = 0.002 z_2_ = 0.89, *p* = 1.000	H = 28.79 *p* < 0.001
HF3%	76.10 (66.00, 82.50)	30.40 (24.60, 43.40) z_1_ = 5.29, *p* < 0.001	36.30 (25.90, 75.30) z_1_ = 3.48, *p* = 0.002 z_2_ = 1.14, *p* = 0.759	H = 31.83 *p* < 0.001
LF/HF3	0.30 (0.20, 0.40	1.50 (1.00, 2.00 z_1_ = 5.31, *p* < 0.001	1.20 (0.30, 2.10 z_1_ = 3.64, *p* < 0.001 z_2_ = 1.02, *p* = 0.922	H = 33.07 *p* < 0.001
LF4%	17.70 (14.08, 25.49)	45.25 (37.80, 49.61) z_1_ = 5.06, *p* < 0.001	42.72 (21.33, 52.64) z_1_ = 3.62, *p* < 0.001 z_2_ = 0.83, *p* = 1.000	H = 30.34 *p* < 0.001
HF4%	80.12 (68.22, 83.96)	31.05 (25.24, 44.67) z_1_ = 5.33, *p* < 0.001	37.65 (26.31, 76.63) z_1_ = 3.54, *p* = 0.001 z_2_ = 1.11, *p* = 0.797	H = 32.50 *p* < 0.001
LF/HF4	0.20 (0.20, 0.40)	1.50 (1.00, 1.90) z_1_ = 5.22, *p* < 0.001	1.10(0.30, 2.10) z_1_ = 3.67, *p* < 0.001 z_2_ = 0.92, *p* = 1.000	H = 32.48 *p* < 0.001
Leg PPGV				
LF1%	32.67 (26.88, 40.13)	44.00 (39.19, 46.87) z_1_ = 3.26, *p* = 0.003	42.57 (32.25, 46.26) z_1_ = 2.08, *p* = 0.112 z_2_ = 0.71, *p* = 1.000	H = 12.34, *p* = 0.002
HF1%	60.94 (51.82, 68.33)	27.60 (17.77, 36.32) z_1_ = 4.35, *p* < 0.001	33.42 (23.45, 58.80) z_1_ = 2.27, *p* = 0.069 z_2_ = 1.38, *p* = 0.498	H = 20.46, *p* < 0.001
LF/HF1	0.52 (0.39, 0.78)	1.86 (1.16, 2.38) z_1_ = 4.16, *p* < 0.001	1.38 (0.53, 1.93) z_1_ = 2.33, *p* = 0.059 z_2_ = 1.87, *p* = 0.706	H = 19.11, *p* < 0.001
LF2%	17.51 (13.36, 26.43)	38.69 (33.20, 43.67) z_1_ = 3.92, *p* < 0.001	35.61 (17.09, 41.14) z_1_ = 2.39, *p* = 0.051 z_2_ = 0.95, *p* = 1.000	H = 17.51 *p* < 0.001
HF2%	79.06 (68.09, 84.46)	37.02 (24.60, 46.77) z_1_ = 4.39, *p* < 0.001	42.53 (35.72, 75.40) z_1_ = 2.38, *p* = 0.052 z_2_ = 1.32, *p* = 0.561	H = 21.02 *p* < 0.001
LF/HF2	0.20 (0.20, 0.40)	1.10 (0.80, 1.50) z_1_ = 4.38, *p* < 0.001	1.00 (0.20, 1.10) z_1_ = 2.39, *p* = 0.051 z_2_ = 1.31, *p* = 0.572	H = 21.56 *p* < 0.001
LF3%	21.60 (17.00, 30.55)	40.20 (35.60, 44.10) z_1_ = 3.83, *p* < 0.001	37.90 (21.30, 43.30) z_1_ = 2.41, *p* = 0.048 z_2_ = 0.87, *p* = 1.000	H = 16.93 *p* < 0.001
HF3%	73.25 (64.65, 79.65)	34.00 (22.00, 43.10) z_1_ = 4.34, *p* < 0.001	38.70 (29.50, 71.90) z_1_ = 2.36, *p* = 0.054 z_2_ = 1.30, *p* = 0.581	H = 20.63 *p* < 0.001
LF/HF3	0.30 (0.20, 0.50)	1.20 (0.90, 1.80) z_1_ = 4.30, *p* < 0.001	1.10 (0.30, 1.40) z_1_ = 2.50, *p* = 0.037 z_2_ = 1.15, *p* = 0.754	H = 21.16 *p* < 0.001
LF4%	19.64 (15.82, 28.99)	39.53 (34.57, 43.89) z_1_ = 3.91, *p* < 0.001	36.83 (19.77, 42.36) z_1_ = 2.39, *p* = 0.049 z_2_ = 0.94, *p* = 1.000	H = 17.42 *p* < 0.001
HF4%	75.88 (66.08, 81.22)	35.02 (22.65, 44.45) z_1_ = 4.35, *p* < 0.001	40.50 (33.29, 73.11) z_1_ = 2.41, *p* = 0.048 z_2_ = 1.27, *p* = 0.611	H = 20.83 *p* < 0.001
LF/HF4	0.30 (0.20, 0.45)	1.20 (0.80, 1.70) z_1_ = 4.37, *p* < 0.001	1.00 (0.30, 1.20) z_1_ = 2.44, *p* = 0.044 z_2_ = 1.25, *p* = 0.630	H = 21.42 *p* < 0.001

Note: z_1_—post hoc analysis of healthy full-term newborns; z_2_—post hoc analysis of full-term newborns with pathology; for indicators that reached statistically significant differences (*p* > 0.05) based on the Kruskal–Wallis test, post hoc analysis was not performed. PPGV—photoplethysmographic waveform variability; HRV—heart rate variability. Time domain indices of HRV: HR—mean heart rate; SDNN—standard deviation of the NN interval (the time elapsing between two consecutive R waves in the electrocardiogram with normal sinus rhythm); RMSSD—square root of the mean squared differences of successive NN intervals; PNN50—proportion derived by dividing NN50, the number of interval differences of successive NN intervals greater than 50 ms, by the total number of NN intervals. Frequency domain indices: LF—power spectral density of low-frequency band in spectrum measured in ms^2^; TP1…4—total power spectral density of spectrum measured in ms^2^ and integrated in 0–0.4 Hz band (named as TP1), 0–0.15 + 0.2–2 Hz band (named as TP2), 0–0.8 Hz band (named as TP3), and 0–0.15 + 0.24–1.04 Hz band (named as TP4); HF1…4—power spectral density of the high-frequency band in the spectrum was measured in ms^2^ and integrated with our accepted boundary options: 0.15–0.40 Hz band (named as HF1), 0.2–2 Hz band (named as HF2), 0.15–0.8 Hz band (named as HF3), and 0.24–1.04 Hz band (named as HF4); LF1…4%—low-frequency band in percentage of total spectral power (TP1…4, consequently); HF1…4%—high-frequency band (HF1…4, consequently) in percentage of total spectral power (TP1…4, consequently); LF/HF1...4—the ratio of low-frequency band to high-frequency band (HF1…4, consequently).

## Data Availability

The data presented in this study are available on request from the corresponding author.

## Supplementary Materials

The following supporting information can be downloaded at: https://www.mdpi.com/article/10.3390/life14060675/s1, Supplement S1. Comparison of assessments of HRV and PPGV spectra recorded on the forehead and leg in groups of full-term newborns with pathology and preterm newborns vs. healthy full-term newborns.
