# Supplementary material for: Full-Term and Preterm Newborns Differ More Significantly in Photoplethysmographic Waveform Variability than Heart Rate Variability
Source: Life (Basel). 2024 May 24;14(6):675. doi: 10.3390/life14060675 (PMC11204696; doi:10.3390/life14060675)
Supplement: Supplementary file 1 [file life-14-00675-s001.zip › life-2997235-supplementary.pdf]

Supplement S1. Comparison of assessments of HRV, respiration, S index and PPGV spectra recorded on the forehead and leg in groups of full-term newborns with pathology and preterm newborns vs. healthy full-term newborns

Color gradation chart is based on strength of differences calculated from the z score and *p*-level in post hoc analyses of healthy subjects:

Color					
z value	–	<3.0	3.0–3.9	4.0–4.9	≥5.0
p-level	*p*>0.05	*p*<0.05			

Statistically significant differences between full-term newborns with pathology and preterm newborns, but none vs. healthy newborns:

Color	
z value	–
p-level	*p*<0.05

Parameters	Healthy Full-term Newborns (Group 1) (n = 64)	Full-term Newborns with Pathology (Group 2) (n = 23)	Preterm Newborns (Group 3) (n = 17)
HR, bpm			↑
Respiratory frequency, Hz			
Respiratory signal range			↓
S (HRV-PPGV forehead), %			
S (HRV-PPGV leg), %			
HRV			
SDNN, ms			
RMSSD			↑
PNN50			↑
LF, ms²			
TP1, ms²			
HF1, ms²			
LF1%			
HF1%			
LF/HF1			
TP2, ms²			
HF2, ms²			↑
LF2%			
HF2%			↑
LF/HF2			↓
TP3, ms²			
HF3, ms²			
LF3%			
HF3%			↑
LF/HF3			↓
TP4, ms²			
HF4, ms²			↑
LF4%			
HF4%			↑
LF/HF4			↓
Forehead PPGV			
LF1%		↑	↑
HF1%		↓	↓
LF/HF1		↑	↑

Parameters	Healthy Full-term Newborns (Group 1) (n = 64)	Full-term Newborns with Pathology (Group 2) (n = 23)	Preterm Newborns (Group 3) (n = 17)
LF2%		↑	↑
HF2%		↓	↓
LF/HF2		↑	↑
LF3%		↑	↑
HF3%		↓	↓
LF/HF3		↑	↑
LF4%		↑	↑
HF4%		↓	↓
LF/HF4		↑	↑
Leg PPGV			
LF1%		↑	↑
HF1%		↓	↓
LF/HF1		↑	↑
LF2%		↑	↑
HF2%		↓	↓
LF/HF2		↑	↑
LF3%		↑	↑
HF3%		↓	↓
LF/HF3		↑	↑
LF4%		↑	↑
HF4%		↓	↓
LF/HF4		↑	↑

Note: PPGV, photoplethysmographic waveform variability; HRV, heart rate variability.

Time domain indices of HRV:

- HR, mean heart rate;
- SDNN, standard deviation of the NN interval (the time elapsing between two consecutive R waves in the electrocardiogram with normal sinus rhythm);
- RMSSD, square root of the mean squared differences of successive NN intervals;
- PNN50, proportion derived by dividing NN50, the number of interval differences of successive NN intervals greater than 50 ms, by the total number of NN intervals.

Frequency domain indices:

- LF, power spectral density of low-frequency band in spectrum measured in ms2;
- TP1…4, total power spectral density of spectrum measured in ms2 and integrated in 0–0.4 Hz band (named as TP1), 0–0.15 + 0.2–2 Hz band (named as TP2), 0–0.8 Hz band (named as TP3), and 0–0.15 + 0.24–1.04 Hz band (named as TP4);
- HF1…4, power spectral density of high-frequency band in spectrum measured in ms2 and integrated our accepted boundary options: 0.15–0.40 Hz band (named as HF1), 0.2–2 Hz band (named as HF2), 0.15–0.8 Hz band (named as HF3), and 0.24–1.04 Hz band (named as HF4);
- LF1…4%, low-frequency band in percentage of total spectral power (TP1…4, consequently);
- HF1…4%, high-frequency band (HF1…4, consequently) in percentage of total spectral power (TP1…4, consequently);
- LF/HF1...4, the ratio of low-frequency band to high-frequency band (HF1…4, consequently).
